# High blood glucose variability may predict poor outcomes in patients with spontaneous cerebellar hemorrhage undergoing surgical operation: a retrospective study

**DOI:** 10.1186/s12883-024-03758-1

**Published:** 2024-07-15

**Authors:** Lei Cheng, Guifeng Yang, Jian Sun, Junwei Ma, Mingchao Fan

**Affiliations:** 1 Department of Neurosurgery, the Affiliated Hospital of Qingdao University, Qingdao, 266003 China; 2 Department of Radiology, the Third People’s Hospital of Qingdao, Qingdao, 266041 China; 3 Department of Neurosurgical Intensive Care Unit, the Affiliated Hospital of Qingdao University, Qingdao, 266003 China

**Keywords:** Spontaneous cerebellar hemorrhage, Blood glucose variability, Hyperglycemia, Surgical operation

## Abstract

**Background:**

Elevated blood glucose (BG) variability has been reported as an independent risk factor for poor prognosis in a variety of diseases. This study aimed to investigate the association between BG variability and clinical outcomes in patients with spontaneous cerebellar hemorrhage (SCH) undergoing surgical operation.

**Methods:**

This retrospective cohort study of the consecutive patients admitted to the department of Neurosurgery, the Affiliated Hospital of Qingdao University between January 2014 and June 2022 with the diagnosis of SCH underwent surgical intervention. BG analysis was continuously and routinely performed. BG variability was represented by the standard deviation (SD) of the serial measurements within the first 7 days. The general characteristics, imageological information, blood glucose level, and surgical information were reviewed and compared through medical records.

**Results:**

A total of 115 patients (65 male and 50 female) were enrolled. Out of all 115 patients, the overall clinical outcomes according to the modified Rankin Scale (mRS) were poor (mRS 3–6) in 31 patients (26.96%) and good (mRS 0–2) in 84 patients (73.04%). Twelve of the 115 patients died during hospitalization, and the mortality rate was 10.43%. Multivariate logistic regression analysis showed that SD of BG (odds ratio (OR), 4.717; 95% confidence interval (CI), 1.054–21.115; *P* = 0.043), GCS (OR, 0.563; 95% CI, 0.330–0.958; *P* = 0.034), and hematoma volume (OR, 1.395; 95% CI, 1.118–1.748; *P* = 0.003) were significant predictors. The area under the ROC curve of SD of BG was 0.911 (95% CI, 0.850–0.973; *P* < 0.001) with a sensitivity and specificity of 90.3% and 83.3%, respectively, and the cut-off value was 1.736.

**Conclusions:**

High BG Variability is independently correlated with the 6-month poor outcomes in patients with SCH undergoing surgical operation.

**Supplementary Information:**

The online version contains supplementary material available at 10.1186/s12883-024-03758-1.

## Introduction

Spontaneous cerebellar hemorrhage (SCH) is a subtype of hemorrhagic stroke, and accounts for 5–10% of all patients with spontaneous intracerebral hemorrhage [1, 2], and hypertension is the common cause [3]. As a relatively closed and narrow space, the posterior fossa lacks adequate compensation for the occupying effect of hematoma. Smaller cerebellar hematoma also tends to cause compression of the brain stem and fourth ventricle, and leads to acute obstructive hydrocephalus and a significant decrease in consciousness [4–6]. Furthermore, the hematoma can break into the ventricular system via the fourth ventricle, induce and aggravate the hydrocephalus [7]. Although the effectiveness of supratentorial hemorrhage is still controversial, the surgical operation of cerebellar hemorrhage has a positive effect on improving the clinical prognosis [2, 8, 9]. Surgical intervention should be considered if brain stem compression and/or acute hydrocephalus attributed to mass effect from hematoma. Compared with supratentorial intracerebral hemorrhage, the pathophysiological mechanisms and treatment strategies of SCH are different, so the risk factors should be analyzed separately [6, 8], but fewer data are available concerning.

Hyperglycemia is often associated with unfavorable outcomes in patients with acute critical illness, as is intracranial hemorrhage [10–12]. Most previous studies have focused on the relationship between absolute blood glucose (BG) levels at admission and clinical outcomes [13, 14]. There are also researcher who reported that BG level at admission was not the independent risk factor for the prognosis of patients with cerebral hemorrhage [15, 16]. Data from recent studies suggest that continuous measurement of BG during hospital stay are more predictive value than a single dose of BG at admission [17, 18]. Several previous investigators reported that Increased BG variability was an independent predictor of worse clinical outcomes in various subgroups critical ill patients, not limited to patients with stroke [12, 19–21]. However, it is not known that the association between increased BG variability and clinical outcomes in SCH patients underwent surgical treatment. The purpose of this study was to investigate the relationship between increased BG variability as reflected by the standard deviation (SD) on clinical outcomes in SCH patients underwent surgical operation.

## Methods

### Study cohort

This study is a retrospective cohort study and is reported following the STROBE guidelines [22]. This retrospective cohort study of the consecutive patients admitted to the department of Neurosurgery, the Affiliated Hospital of Qingdao University between January 2014 and June 2022 with the diagnosis of SCH underwent surgical intervention. SCH was diagnosed by two senior neurosurgeons and one radiologist based on history of present illness, neuroimaging, and surgical operation findings.

Inclusion criteria were: aged ≥ 18 year-old; diagnosed with SCH; underwent surgical operation treatment; hospital stay more than 7 days.

Exclusion criteria were: with a history of craniotomy, traumatic brain injury or stroke; secondary cerebellar hemorrhage; incomplete information; loss of follow-up.

### Clinical data collection

Clinical information during hospitalization was collected from the information system and the scientific research big data platform of our hospital. Basic population information, such as gender, age, weight, height, past medical history, and personal life history were registered. Vital signs were monitored and recorded. The level of consciousness was assessed and quantified with the Glasgow Coma Scale (GCS) [23]. The blood routine analysis, blood biochemical analysis and hemagglutination were performed immediately on admission.

BG was measured immediately upon admission, and hyperglycemia was defined as fasting BG levels on admission ≥ 6.9mmol/L. Insulin was administered and adjusted for patients whose BG was ≥ 10mmol/L, and control the BG between 7 and 9 mmol/L. Fingertip blood glucose testing was performed regularly. BG tests were routinely performed 3 times a day, and 6 times (about 06:30, 09:30, 12:30, 15:30, 18:30 and 21:30) if the BG tests were abnormal during hospitalization. The laboratory testing was not performed during sleep at night, unless the condition changes, so as not to affect patients’ rest. For each patient enrolled in the study, the mean BG concentration and SD were calculated. The variability of BG levels in this patient was reflected by SD values.

Brain CT scanning was performed within 2 h at admission for all patients. The angiography and/or magnetic resonance imaging (MRI) scans were performed if the cause of the bleeding was suspicious. The location and size of the hematoma and intraventricular hemorrhage were evaluated. The hematoma volume was calculated using the hematoma volume calculation formula: “0.5 × a × b × c”, where ‘a’ and ‘b’ are the diameters of the largest section measured by CT scan, and ‘c’ is the CT scan thickness (cm), was used to calculate the volume of the hematoma [24]. The main hematoma location was categorized into vermis, cerebellar hemispheric or hemispheres + vermis. Based on the condition and imaging characteristics, the neurosurgeon determined the surgical method with the consent of the patient’s guardians. The indications and procedures of operation are performed in accordance with the corresponding surgical guidelines and conventional surgical procedures [7, 25], and necessary adjustments were made according to the characteristics of the patient. Every patient receives standard and necessary medical care. Admission to the neurosurgical intensive care unit and the length of stay was determined by consensus between the neurocritical care physician and neurosurgeon based on the state and needs of each patient.

The routine follow-up was performed at the neurosurgery clinic, and the telephone interviews was also accepted if the patient put off checking-up or has limited mobility [26–28]. Patients who were lost to follow-up and died from other unrelated causes were excluded. The modified Rankin Scale (mRS) was used to evaluate the prognosis [29]. The prognosis was evaluated at 6-month after onset. All Patients were divided into two groups based on mRS: good prognosis group (mRS 0–2) and poor prognosis group (mRS 3–6).

### Statistical analysis

IBM SPSS Statistics 24.0 (SPSS Inc., Chicago, Illinois, USA) was used to statistical analysis. It is estimated that the sample size of this study meets the requirements. The means ± SD was used to shown the normal distribution variables. The median and interquartile ranges (25th to 75th percentile) were used to represent the abnormal distribution of continuous variables. Categorical variables were presented as frequency and percentage. Student’s *t* test was used to compare the continuous variables of normal distribution. The Kruskal-Wallis test was used to compare the abnormal distribution continuous variables. The Chi-square test was used to compare the categorical data variables. The logistic regression analyses was conducted to build a prediction model. Receiver operating characteristic curve (ROC) analysis was performed to demonstrate the predictive value of the parameters, compare the area under the curves (AUC) and define the cutoff value. The difference was statistically significant in *P* value < 0.05.

## Results

**Fig. 1:**
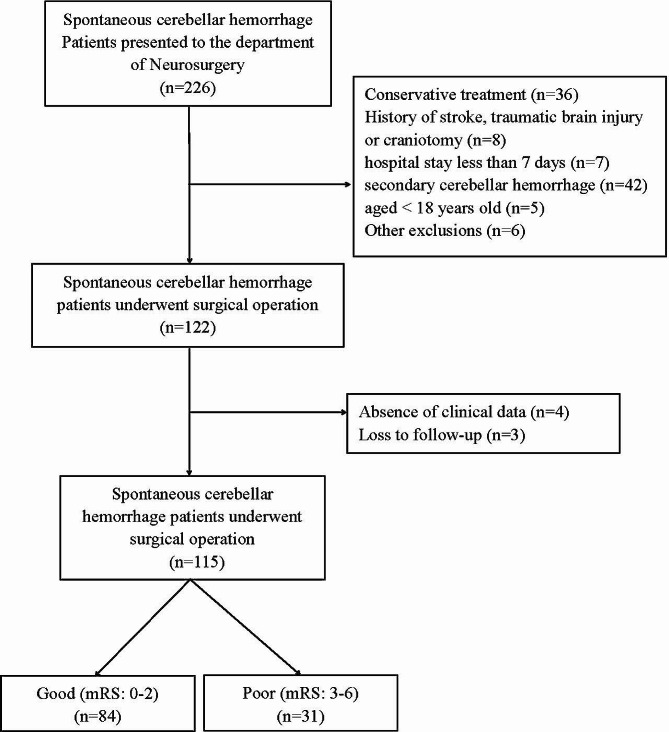
The study flow chart

The demographics’ information of all patients is presented in Table 1. A total of 115 consecutive patients with SCH underwent surgical operation were enrolled. (Fig. 1). Of these, 84 patients (73.04%) had good outcomes (mRS 0–2) and 31 (26.96%) had poor outcomes (mRS 3–6) at 6 months after attack. Twelve of the 115 patients died during hospitalization, and the mortality rate was 10.43%. Sixty-five of the 115 patients (56.52%) were male. The median age was 63 years (interquartile range, 54–71 years).

**Table 1:** The demographic and baseline characteristics

Variable	Total (*n* = 115)	6-month prognosis		*P*-value
		Favorable (*n* = 84)	Unfavorable (*n* = 31)	
Sex (male, %)	65 (56.52)	49 (58.33)	16 (51.61)	0.519
Age (years)	63.00 (54.00–71.00)	63.00 (56.25–70.75)	64.00 (42.00–75.00)	0.377
BMI	24.20 (21.50–26.70)	24.20 (21.53–26.65)	23.90 (21.50–27.20)	0.845
Smoking (%)	22 (19.13)	17 (20.24)	5 (16.13)	0.619
Drinking (%)	17 (14.78)	12 (14.29)	5 (16.13)	0.774
Hypertension (%)	73 (63.48)	52 (61.90)	21 (67.74)	0.564
Cardiac insufficiency (%)	8 (6.96)	7 (8.33)	1 (3.23)	0.311
History of Diabetes	15 (13.04)	8 (9.52)	7 (22.58)	0.125
Systolic Pressure (mmHg)	156.00 (140.00-176.00)	154.00 (136.25–171.50)	162.00 (145.00-180.00)	0.282
Diastolic Pressure (mmHg)	91.00 (82.00-102.00)	90.50 (82.25–100.00)	91.00 (81.00-105.00)	0.508
Heart rate (%)	84.00 (76.00–90.00)	80.00 (76.00–90.00)	86.00 (78.00–98.00)	0.157
Length of ICU stay (days)	0.00 (0.00–4.00)	0.00 (0.00–1.00)	10.00 (10.00–18.00)	**< 0.001**
Length of stay (days)	14.00 (10.00–20.00)	13.00 (9.25–18.75)	18.00 (11.00–27.00)	**0.015**
GCS on admission	14.00 (9.00–15.00)	15.00 (13.00–15.00)	6.00 (4.00–9.00)	**< 0.001**
Ventricular hematocele (%)	47 (40.87)	30 (35.71)	17 (54.84)	0.064
Hydrocephalus (%)	61 (53.04)	36 (42.86)	25 (80.65)	**< 0.001**
Hematoma volume (mL)	10.00 (10.00–20.00)	10.00 (10.00–15.00)	25.00 (20.00–30.00)	**< 0.001**
Hematoma location (%) Hematoma location				0.916
Left hemispheres	55 (47.80)	39 (46.43)	16 (51.61)	
Right hemispheres	38 (33.00)	28 (33.33)	10 (32.26)	
Left hemispheres + vermis	10 (8.70)	7 (8.33)	3 (9.68)	
Right hemispheres + vermis	7 (6.10)	6 (7.14)	1 (3.23)	
Left and right hemispheres + vermis	5 (4.30)	4 (4.76)	1 (3.23)	
Surgical approach (%)				0.496
Craniotomy	72 (62.61)	55 (65.48)	17 (54.84)	
Hematoma drainage	10 (8.70)	6 (7.14)	4 (12.90)	
Lateral ventricle catheterization	33 (28.70)	23 (27.38)	10 (32.26)	
WBCs (×10^9^/L)	11.90 (10.13–14.84)	11.66 (9.58–14.65)	13.53 (11.29–15.02)	0.069
Hemoglobin (g/L)	146.00 (136.00-158.00)	145.50 (136.25–157.50)	146.00 (136.00-159.00)	0.885
Platelet (×10^9^/L)	203.00 (176.00-255.00)	197.50 (174.50-251.75)	207.00 (185.00-267.00)	0.301
Serum albumin (g/L)	39.40 (36.55–42.47)	39.80 (36.70-42.85)	38.30 (34.00-41.70)	0.057
Creatinine (umol/L)	62.00 (51.20–72.00)	60.55 (50.23–70.60)	67.70 (53.40-77.14)	0.155
Uric acid (µmol/L)	230.00 (174.10-286.10)	224.40 (173.28-274.95)	237.40 (178.00-310.00)	0.516
Total bilirubin (umol/L)	15.24 (12.00-19.38)	15.22 (12.06–19.70)	15.30 (10.40–18.80)	0.630
Triglyceride (mmol/L)	1.16 (0.78–1.53)	1.22 (0.78–1.67)	0.99 (0.63–1.29)	0.073
Fibrinogen (mmol/L)	3.10 (2.52–3.59)	3.05 (2.52–3.49)	3.15 (2.56–3.73)	0.664
D-dimer (µg/L)•	461.00 (290.00-800.00)	455.00 (310.00-840.00)	470.00 (270.00-770.00)	0.907
Serum K^+^ (mmol/L)	3.84 (3.50–4.10)	3.90 (3.50–4.10)	3.60 (3.49–4.20)	0.155
Serum Na^+^ (mmol/L)	140.55 (138.00-142.53)	140.25 (138.00-142.23)	141.05 (137.75-143.33)	0.394
BG on admission (mmol/L)•	7.98 (6.30-10.08)	7.05 (6.12–8.65)	11.30 (9.10–14.20)	**< 0.001**
Mean of BG (mmol/L)	6.84 (6.09–8.13)	6.85 (5.94–8.07)	6.77 (6.24–8.15)	0.640
SD of BG	1.22 (0.69–2.17)	0.96 (0.59–1.43)	2.26 (2.03–3.96)	**< 0.001**

BMI, body mass index; GCS, Glasgow coma scale; BG, ; WBCs, white blood cells; SD, K^+^, potassium ion; Na^+^, sodion

The significance of bold data: *P* < 0.05

**Fig. 2:**
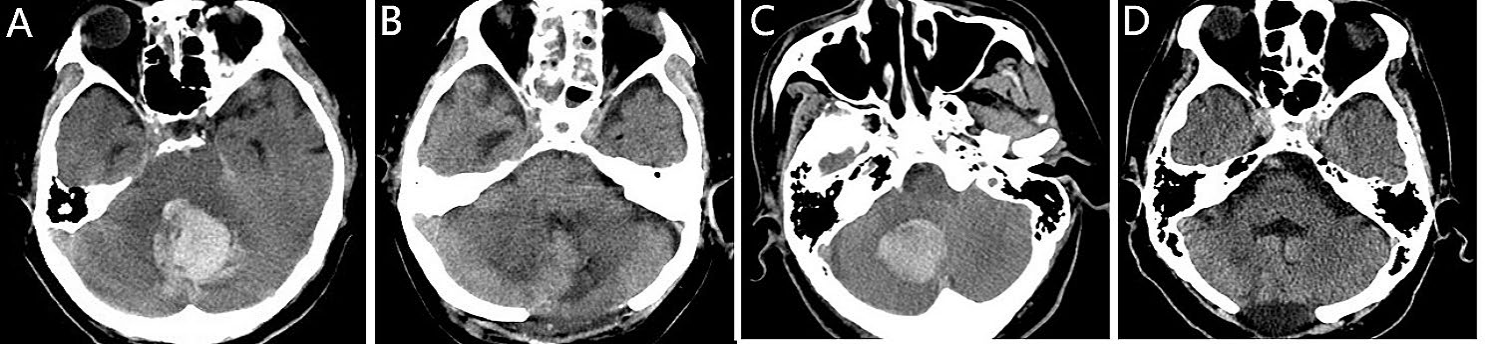
**A&B**: A 56-year-old male patients with cerebellar hematoma and the CT morphology of the posterior cranial fossa after craniotomy. **C&D**: A 62-year-old female patients with cerebellar hematoma and the CT morphology of the fourth ventricle and cisterna ambiens after craniotomy

Of the 115 patients, 55 had cerebellar hematoma mainly located in the left cerebellar hemisphere, 38 patients in the right, 10 patients in the left hemisphere and vermis, 7 patients in the right hemisphere and vermis, and 5 patients in the left and right hemispheres and vermis. (Fig. 2) The difference among different hematoma locations correlated with the 6-month prognosis had no statistical significance (*P* = 0.496). Forty-seven patients (40.87%) had Ventricular hematocele, but there was no significant effect on 6-month clinical prognosis (*P* = 0.064). All patients underwent surgical operation; 72 patients (62.61%) underwent craniotomy, 10 patients (8.70%) underwent hematoma drainage; and 33 patients (28.70%) underwent lateral ventricle drainage. The difference among different surgical operations correlated with the 6-month prognosis had no statistical significance (*P* = 0.496). The total hospital stay total hospital stay and total hospital stay in the favorable group were significantly shorter than those in the unfavorable group (18 days vs. 13 days, *P* = 0.015; 10 day vs. 0 days, *P* < 0.001; respectively).

### Univariate and multivariate logistic regression analyses

The results of univariate and multivariate logistic regression analyses are shown in Table 2. Univariate analysis revealed that GCS (odds ratio (OR), 0.493; 95% confidence interval (CI), 0.380–0.641; *P* < 0.001), hydrocephalus (OR, 5.556; 95%CI, 2.064–14.955; *P* = 0.001), hematoma volume (OR, 1.493; 95%CI, 1.277–1.745; *P* < 0.001), BG on admission (OR, 1.752; 95%CI, 1.399–2.193; *P* < 0.001), and SD of BG (OR, 6.977; 95%CI, 3.317–14.675; *P* < 0.001) were significantly correlated with the 6-month prognosis.

**Table 2:** Univariate and multivariate regression analysis of factors related to prognosis

Predictors	Univariate analysis		Multivariate analysis	
	OR (95% CI)	*P*-value	OR (95% CI)	*P*-value
GCS on admission	0.493 (0.380–0.641)	< 0.001	**0.563 (0.330–0.958)**	**0.034**
Hydrocephalus	5.556 (2.064–14.955)	0.001	0.181 (0.009–3.834)	0.272
Hematoma volume	1.493 (1.277–1.745)	< 0.001	**1.398 (1.118–1.748)**	**0.003**
BG on admission	1.752 (1.399–2.193)	< 0.001	0.903 (0.517–1.577)	0.719
SD of BG	6.977 (3.317–14.675)	< 0.001	**4.717 (1.054–21.115)**	**0.043**

GCS, Glasgow coma scale; PNI, prognostic nutritional index; WBCs, white blood cells; RBCs, red blood cells, K^+^, potassium ion

The significance of bold data: *P* < 0.05

These significant factors were further analyzed with multivariate logistic regression analysis. The SD of BG (OR, 4.717; 95% CI, 1.054–21.115; *P* = 0.043), GCS (OR, 0.563; 95% CI, 0.330–0.958; *P* = 0.034), and hematoma volume (OR, 1.395; 95% CI, 1.118–1.748; *P* = 0.003) were found to be associated with 6-month prognosis after onset.

### ROC curve analysis

**Fig. 3:**
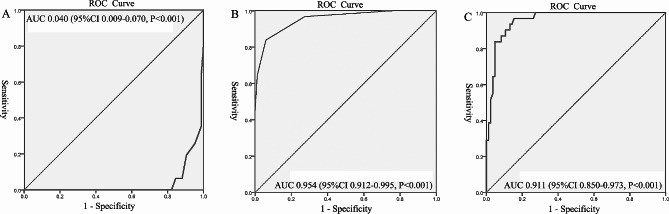
The receiver-operating characteristic curves. **(A)** ROC for GCS; **(B)** ROC for Hematoma volume; **(D)** ROC for SD of BG. (ROC, Receiver Operating Characteristics Curve; GCS, Glasgow Coma Scale; SD: standard deviation; BG, blood glucose.)

The predictive ability of GCS, hematoma volume, and SD of BG was demonstrated using ROC curves (Fig. 3; Table 3). The corresponding AUC of GCS was 0.040 (95% CI, 0.009–0.070, *P* < 0.001), and the cutoff value was 12.5, with a specificity and sensitivity of 82.1% and 100%, respectively. The ROC curve AUC of hematoma volume was 0.954 (95% CI, 0.912–0.995, *P* < 0.001), and the cutoff value was 17.5, with a specificity and sensitivity of 94.0% and 83.9%, respectively. The ROC curve AUC of SD of BG was 0.911 (95% CI, 0.850–0.973, *P* < 0.001), and the cutoff value was 1.736, with a specificity and sensitivity of 83.3% and 90.3%, respectively.

**Table 3:** Diagnostic values of factors related to poor prognosis

Variable	AUC (95% CI)	*P*-value	Cutoff Value	Sensitivity	Specificity
GCS on admission	0.040 (0.009–0.070)	**< 0.001**	12.5	1.000	0.821
Hematoma volume	0.954 (0.912–0.995)	**< 0.001**	17.500	0.839	0.940
SD of BG	0.911 (0.850–0.973)	**< 0.001**	1.736	0.903	0.833

PNI, prognostic nutritional index

The significance of bold data: *P* < 0.05

## Discussion

We retrospective retrieved and analyzed the clinical data from SCH patients who underwent surgical operation in our neurosurgical center during an 6-year period. The multivariate logistic regression analysis and ROC curve analysis were used to determine the predictors. We found that GCS, hematoma volume, and SD of BG were important 6-month predictors in SCH patients who underwent surgical operation. GCS and hematoma volume are the conventional useful prognostic indicators of SCH, while SD of BG is a new indicator. These results suggest that BG variability is an independent risk marker for SCH patients underwent surgical treatment. As far as we know, we showed that the SD of BG is independently correlated with the 6-month prognosis in SCH patients underwent surgical operation in this study for the first time. These findings may be useful for the doctors and nurses to assess the condition and therapeutic regimen. At the same time, help the patients’ families to understand the severity of the disease, in order to adjust their role to face the challenges of life.

BG variability reflects the tendency of BG levels to fluctuate, and is considered as a third indicator of dysglycemia apart from hypoglycemia and hyperglycemia [21, 30, 31]. The basis of increased BG variability is high blood glucose levels in patients with acute stroke. Post-stroke hyperglycemia is mainly attributed to stress hyperglycemia, diabetes with poor control, and stress elevation of BG on the basis of diabetes. Stress hyperglycemia after acute stroke, especially hemorrhagic stroke, has strong associations with poor functional recovery and high mortality [32–34]. Stress hyperglycemia after acute stroke reduces the level of energy metabolism of brain microvascular endothelial cells and aggravates the destruction in blood-brain barrier by inhibiting mitochondrial function [35]. Vascular endothelial cell injury and increased blood-brain barrier permeability promote the progression of vasogenic brain edema around the hematoma, ultimately leading to neuron death [36]. Tao et al. [8] retrospectively analyzed the relationship between BG level at admission and 6-month clinical outcome in 77 patients with cerebellar hemorrhage, and found a significant negative correlation between high BG level at admission and 6-month clinical outcome through univariate and multivariate logistic regression analysis. In this current study, BG levels at admission were associated with a 6-month clinical prognosis only in univariate analysis, but no positive findings were found in multivariate logistic regression analysis, similar to the results of Chang et al. [6].

Stroke triggers stressful events activated by the sympathetic adrenal medulla axis and the hypothalamic-pituitary-adrenal axis, where neuroendocrine hormones promote glucagon secretion, inhibit insulin release, induce insulin resistance, stimulate glycogenolysis and gluconogenesis, resulting in elevated BG levels [29, 37]. In this mechanism, elevated post-stroke BG levels were significantly positively associated with stroke severity [8]. Stress hyperglycemia not only refers to reactive BG increases in non-diabetic patients, but also includes stress BG increases in diabetic patients. In addition, acute stroke leads to disability of patients, and those patients with a history of diabetes fall behind in regular hypoglycemic treatment, which is also one of the reasons for hyperglycemia at the time of visit, and needs to be identified by medical personnel.

Elevated BG variability levels can be attributed not only to episodes of hyperglycaemia, but also to hypoglycemic adverse reactions induced by hyperglycemic drug therapy [18]. The use of intensive insulin therapy to control BG levels has been widely adopted for stress hyperglycemia in the last decade [38]. At the same time, intensive insulin therapy for hyperglycemia also faces a lot of opposition, and the high incidence of hypoglycemia is a negative factor [31]. In order to avoid the occurrence of malignant hypoglycemia events, modified intensive insulin therapy for stress hyperglycemia is gradually accepted. We adopted the modified insulin therapy strategy to control the BG in the moderately high range and avoid the occurrence of hypoglycemia as much as possible. Therefore, the negative association between hyperglycemic variants and poor outcomes in our cohort may not be fully explained by episodic hypoglycemia, at least with a small effect. Short-term stress hyperglycemia induced by hemorrhagic stroke and neurosurgical operational trauma may be the main component of increased BG variability.

High BG variability is considered to be potentially related to aggravated oxidative stress. Recently, oxidative stress and inflammatory response have been increasingly recognized as important factors for the secondary brain damage after stroke [39, 40]. Although sustained hyperglycemia promotes the expression of markers of oxidative stress, a fluctuating rise in BG corresponds to a more severe oxidative stress response [13, 41]. Oxidative stress and inflammatory responses lead to impairment of endothelial function by increasing apoptosis of vascular endothelial cells [20, 30]. And fibroblasts produced increased cytokines in cells with fluctuating glucose levels [42], which may increase the inflammatory response. Increased short-term BG fluctuations are thought to damage the innate immune component and increase the risk of infection [43]. Ying et al. [44] established Sprague-Dawley rats blood glucose fluctuation models to analyze the damage to BG variability on cardiomyocytes. And they found that higher BG variability can aggravate of TNF-α levels, and may promote oxidative stress by inhibiting the AKT signaling path, leading to cardiac tissue fibrosis.

Excessive fluctuation on BG level has a severe poor impact on mitochondrial activity of neuronal cells, resulting in mitochondrial stress and significant expression of apoptosis genes [45]. Okazaki et al. [46] reported the effect of increased BG variability on poor neurological prognosis in patients with aneurysmal subarachnoid hemorrhage. In their study, the rate of adverse neurological prognosis increased significantly with the SD of BG. Kurtz et al. [47] retrospectively analyzed the data of patients with subarachnoid hemorrhage, and found that increased BG variability was associated with brain oxygen metabolism disorders and increased mortality. Then, they speculated that reducing BG variability could improve human brain tissue metabolism and prognosis in patients with subarachnoid hemorrhagic.

For the first time, we report BG variability as an independent predictor of 6-month prognosis in SCH patients undergoing neurosurgical operation. Our study has limitations that should be mentioned. First, it is a small, retrospective study done in one single center. Second, Only those patients who received surgical treatment were retrospectively analyzed, and patients receiving conservative treatment in neurology and neurosurgery were excluded, this may have partially limited the range of results. Third, although some patients used corticosteroids in the short-term for brain edema, the effects of corticosteroids on BG levels were not analyzed. Fourth, due to the limitations of the retrospective study, we do not have sufficient data to confirm the specific occurrence of stress hyperglycemia or the data of patients with underlying diabetes. Fifth, as a clinical observational study, specific pathological mechanisms cannot be provided. The multicenter, prospective, and randomized controlled clinical studies should be conducted to further understand BG variability and provide clinical countermeasures.

## Conclusion

High BG Variability is independently correlated with the 6-month poor outcomes in patients with SCH undergoing surgical operation.

### Electronic supplementary material

Below is the link to the electronic supplementary material.

Supplementary Material 1

## Data Availability

The data and code that support the findings of this study are available from the corresponding author upon reasonable request.
